# Supplementary material for: The interactome of intact mitochondria by cross-linking mass spectrometry provides evidence for coexisting respiratory supercomplexes
Source: Mol Cell Proteomics. 2017 Dec 8;17(2):216–32. doi: 10.1074/mcp.RA117.000470 (PMC5795388; doi:10.1074/mcp.RA117.000470)
Supplement: Supplemental Data [file supp_RA117.000470_133922_0_supp_23978_fzffwf.zip › spectra_annotation/mito_spectra_annotation/303-1-6-1-3-1.pdf]

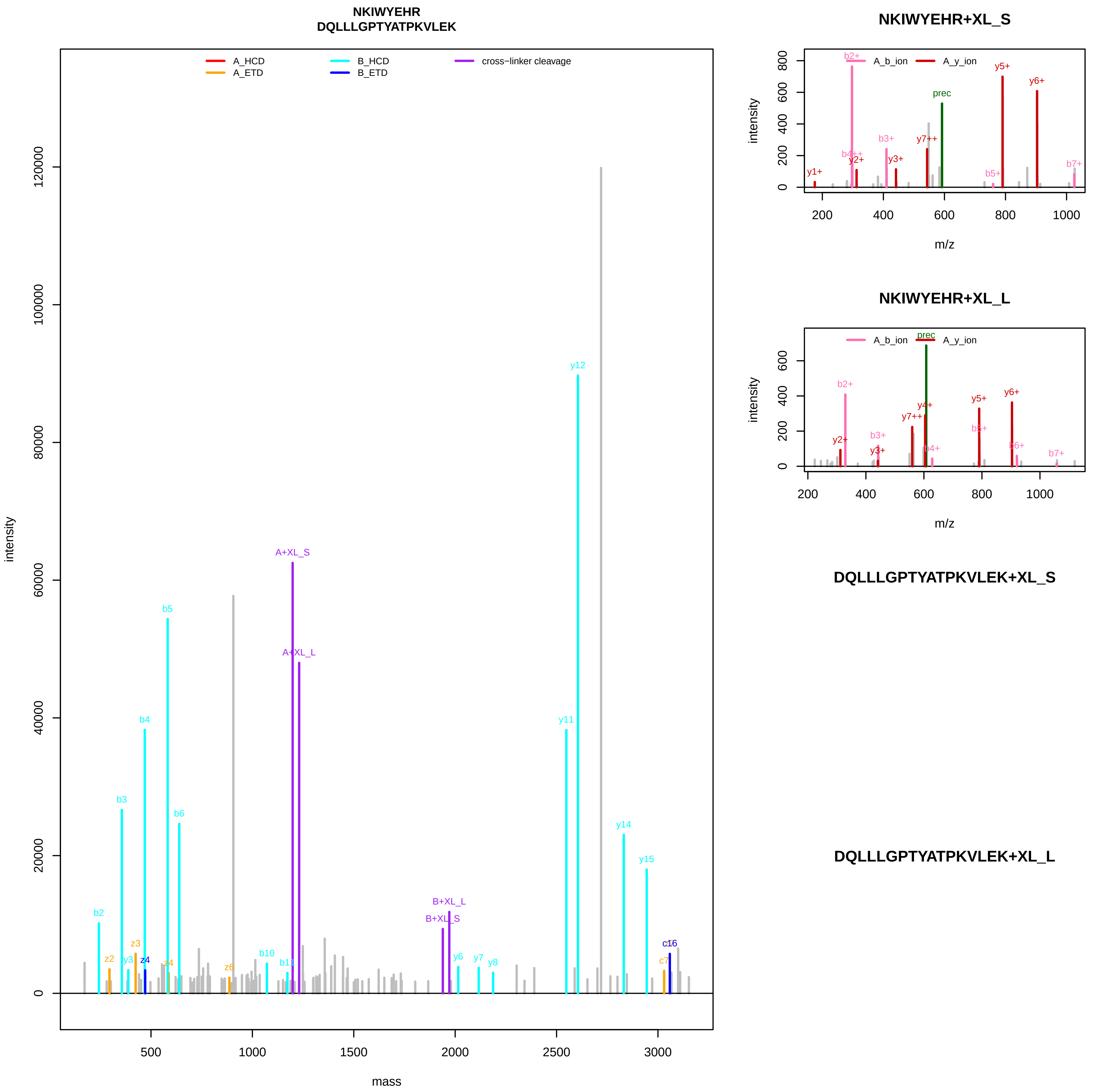

NKIWYEHR
DQLLLGPTYATPKVLEK
A_HCD
A_ETD
B_HCD
B_ETD
cross−linker cleavage
intensity
mass
0
20000
40000
60000
80000
100000
120000
500
1000
1500
2000
2500
3000
b2
b3
b4
b5
b6
b10
b11
y3
y6
y7
y8
y11
y12
y14
y15
z2
z3
z4
z6
c7
c16
A+XL_S
A+XL_L
B+XL_S
B+XL_L
NKIWYEHR+XL_S
A_b_ion
A_y_ion
prec
y1+
y2+
y3+
y5+
y6+
y7++
b2+
b3+
b4++
b5+
b7+
m/z
200
400
600
800
1000
NKIWYEHR+XL_L
y4+
b4+
b6+
DQLLLGPTYATPKVLEK+XL_S
DQLLLGPTYATPKVLEK+XL_L
